# Rediscovery of *Angiopteris tonkinensis* (Marattiaceae) after 100 years, and its revision

**DOI:** 10.3897/phytokeys.161.54912

**Published:** 2020-09-15

**Authors:** Ting Wang, Bo Xiao, En-De Liu, Khang Sinh Nguyen, Jie-Qiu Duan, Kang-Lin Wang, Yue-Hong Yan, Jian-Ying Xiang

**Affiliations:** 1 Southwest Forestry University, College of Biodiversity Conservation, Kunming 650224, China; 2 Southwest Forestry University, Yunnan Academy of Biodiversity, Kunming 650224, China; 3 Shanghai Chenshan Plant Science Research Centre, Chinese Academy of Sciences, Chenshan Botanical Garden, Shanghai 201602, China; 4 Forest Bureau of Malipo County, Malipo 663600, China; 5 Key Laboratory of Biodiversity and Biogeography, Kunming Institute of Botany, Chinese Academy of Sciences, Kunming 650204, China; 6 Institute of Ecology and Biological Resources, Vietnam Academy of Sciences and Technology,18 Hoang Quoc Viet, Nghia Do, Cau Giay, Hanoi, 100000, Vietnam; 7 Southwest Forestry University, College of life Science, Kunming 650224, China; 8 Southwest Forestry University, Green Development Institute, Kunming 650224, China

**Keywords:** *Archangiopteris tamdaoensis*, fern phylogeny, morphology, *Protomarattia tonkinensis*, taxonomy

## Abstract

The border area between south-eastern Yunnan, China and northern Vietnam is one of the regions with richest biological diversity including that of the fern genus *Angiopteris* (Marattiaceae). Based on the analysis of morphology and DNA sequences of multiple chloroplast regions (*atpB*, *rbcL*, *rps4-trnS* spacer and *trnL-F* spacer), we revised *Angiopteris tonkinensis* (Hayata) J.M.Camus and proposed a new combination *Angiopteris tamdaoensis* (Hayata) J.Y.Xiang & T.Wang, **comb. nov.**, which was previously regarded as a synonym of *A. tonkinensis*. We found support for a monophyletic *Angiopteris* including *Protomarattia*. This discovery adds two new distribution sites of *A. tonkinensis*, one in China (Malipo, Yunnan) and one in Vietnam (Quan Ba, Ha Giang). We suggest *A. tonkinensis* should be categorised as Critically Endangered (CR) species according to the criteria of IUCN.

## Introduction

The fern genus *Angiopteris*Hoffmann (1796) comprises about 30–40 species in the world and 28 species (17 endemic) in China (He and Christenhusz 2013). The border area between south-eastern Yunnan, China and northern Vietnam is one of the regions with the richest biological diversity including that of *Angiopteris*. According to He and Christenhusz (2013), there are 16 species (five endemic) in this area. The endemic species in this area include *Angiopteris bipinnata* (Ching) J.M.Camus (Camus 1989), *A. dianyuecola* Z.R.He & W.M.Chu (He and Chu 2006), *A. latipinna* (Ching) Z.R.He, W.M.Chu & Christenh. (He and Christenhusz 2013), *A. sparsisora* Ching (Ching 1982) and *A. subrotundata* (Ching) Z.R.He & Christenhusz (He and Christenhusz 2013).

**Figure 1.**
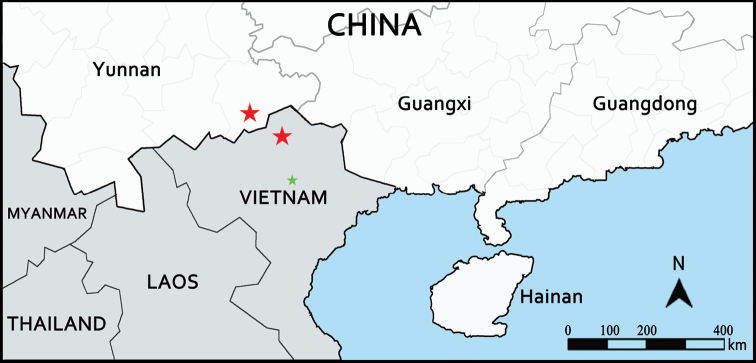
Distribution records of *Protomarattia tonkinensis* Hayata noted by Hayata (1919, green star) and new records in China and Vietnam (red stars).

During our fieldwork in Malipo (south-western China) on 18 May 2018 and Quan Ba (northern Vietnam) on 10 Oct 2019, two small populations of ferns caught our attention (Fig. 1). We identified them as *Protomarattia tonkinensis*Hayata (1919), a very rare species known only from the type specimen collected in Monte Tamdao (Tonkin) of Vietnam and that had never been recorded again since 1919.

*Protomarattia*Hayata (1919), a monotypic genus of Marattiaceae endemic to the Vietnam, was described based on *Protomarattia tonkinensis*. The author pointed out that it differs from its morphologically-similar species, *Archangiopteris tamdaoensis*Hayata (1919) by elongated linear synangia. Based on the morphological study on the isotype of *Protomarattia tonkinensis*, Christensen and Tardieu-Blot (1935) suggested that ‘the sori are young, pressed against each other, compressed, but not fused into ‘synangia’” and treated *P. tonkinensis* as a synonym of *Ar. tamdaoensis.* Later, Ching (1958) treated *P. tonkinensis* and *Ar. tamdaoensis* as synonyms of *Ar. tonkinensis* (Hayata) Ching (1958). Based on previous studies and her own morphological study, Camus (1989) merged *Protomarattia* and *Archangiopteris* into *Angiopteris* and transferred *Ar. tonkinensis* to *Angiopteris* under the new combination *Angiopteris tonkinensis* (Hayata) J.M.Camus (1989). In the modern phylogenetic era, Camus’ (1989) treatment has generally been accepted by many researchers (Murdock 2008a, b; He and Christenhusz 2013; Yan and Zhou 2018; Tropicos (www.tropicos.org)).

In this study, we aimed to explore the identity of the materials collected from Malipo, Yunnan, China and Quan Ba, Ha Giang, Vietnam by means of morphological and phylogenetic studies. We inferred the phylogeny of *Protomarattia tonkinensis* and *Archangiopteris tamdaoensis* based on four chloroplast regions (*atpB*, *rbcL*, *rps4-trnS* and *trnL-F*), then we revised their taxonomic status.

## Material and methods

### Morphological analysis

The Voucher specimens were deposited at Southwest Forestry University (SWFU), the herbarium of Institute of Ecology and Biological Resources, Hanoi, Vietnam (HN) and Shanghai Chenshan Botanical Garden Herbarium (CSH). For morphological comparisons, primary literature (Hayata 1919) and the specimens were critically checked. Petiole scales were observed with a Nikon ECLIPES E100 biological microscope. Sporangia and venation of *Protomarattia tonkinensis* were observed with a LEICA M165 FC stereoscopic fluorescence microscope. The ornamentation of spores was observed with a ZEISS electron scanning microscope.

### Phylogenetic analysis

We analysed 19 samples of Marattiaceae with DNA sequences of four chloroplast regions (*atpB*, *rbcL*, *rps4-trnS* spacer and *trnL-F* spacer). Fourteen sequences of seven species were newly generated for this study and their voucher information and GenBank accession numbers are presented in Table 1. Additional sequences of five species were downloaded from GenBank and their GenBank accession numbers are presented in Table 2.

**Table 1.** Details o.f material newly generated in our study.

Species	Locality	Voucher	GenBank accession number			
			*atpB*	*rbcL*	*rps4-trnS*	*trnL-F*
*Protomarattia tonkinensis* Hayata	Malipo, Yunnan, China	BX19001 (SWFU)	MT855986	MT856000	MT856014	MT856028
*Protomarattia tonkinensis* Hayata	Malipo, Yunnan, China	BX19002 (SWFU)	MT855987	MT856001	MT856015	MT856029
*Protomarattia tonkinensis* Hayata	Malipo, Yunnan, China	BX19003 (SWFU)	MT855988	MT856002	MT856016	MT856030
*Protomarattia tonkinensis* Hayata	Quan Ba, Ha Giang, Vietnam	AT1 (SWFU, HN)	MT855989	MT856003	MT856017	MT856031
*Protomarattia tonkinensis* Hayata	Quan Ba, Ha Giang, Vietnam	AT2 (SWFU)	MT855990	MT856004	MT856018	MT856032
*Protomarattia tonkinensis* Hayata	Quan Ba, Ha Giang, Vietnam	AT4 (SWFU)	MT855991	MT856005	MT856019	MT856033
*Protomarattia tonkinensis* Hayata	Quan Ba, Ha Giang, Vietnam	AT5 (SWFU)	MT855992	MT856006	MT856020	MT856034
*Protomarattia tonkinensis* Hayata	Quan Ba, Ha Giang, Vietnam	AT6 (SWFU)	MT855993	MT856007	MT856021	MT856035
*Archangiopteris tamdaoensis* Hayata	Nankai, Hainan, China	SG2765 (CSH)	MT855994	MT856008	MT856022	MT856036
*Angiopteris bipinnata* (Ching) J.M.Camus	Malipo, Yunnan, China	XJY19001 (SWFU)	MT855995	MT856009	MT856023	MT856037
*Angiopteris chingii* J.M.Camus	Hekou, Yunnan, China	XJY2186 (SWFU)	MT855996	MT856010	MT856024	MT856038
*Angiopteris dianyuecola* Z.R.He & W.M.Chu	Hekou, Yunnan, China	WT19002 (SWFU)	MT855997	MT856011	MT856025	MT856039
*Angiopteris hokouensis* Ching	Hekou, Yunnan, China	WT19001 (SWFU)	MT855998	MT856012	MT856026	MT856040
*Christensenia aesculifolia* (Blume) Maxon	Hekou, Yunnan, China	Zhang Guil456 (SWFU)	MT855999	MT856013	MT856027	MT856041

**Table 2.** Details of material downloaded from GenBank and their accession numbers.

Species	Genbank accession number		
	*rps4-trnS*	*atpB*	*rbcL*
*Marattia alata* Sw.	EU439108	EU439060	EU439082
*Ptisana fraxinea* (Sm.) Murdock.	EU439131	EU439067	EU439088
*Ptisana purpurascens* (de Vriese) Murdock	EU439132	EU439068	EU439089
*Ptisana melanesica* (Kuhn) Murdock	EU439134	EU439069	EU439090
*Ptisana salicina* (Sm.) Murdock	EU439113	EU439063	EU439085

Total genomic DNA was extracted from silica-gel dried leaves using the TSINGKE plant DNA extraction kit (generic). The sequences were amplified using the primers designed by previous studies: primers e and f for *trnL-F* gene (Li and Lu 2006), F1 and R4 for *rbcL* gene (Murdock 2008a), rps5’ (Nadot et al. 1994) and *trnS* R (Smith and Cranfill 2002) for *rps4-trnS*, atpB-F1 (Murdock 2008a) and atpE 384R (Wolf 1997) for *atpB*. Sequencing was performed using the ABI 3730XL DNA analyser (Applied Biological Systems, Foster City, CA, USA).

Sequences were assembled and edited with SeqMan (Burland 1999) and then aligned and manually adjusted on Mega7.0 (Kumar et al. 2016). To estimate phylogenetic relationships, we applied Maximum Likelihood (ML) analysis with concatenated DNA datasets. PartitionFinder2 (Lanfear et al. 2016) was used to select a subset scheme and substitution models as assessed by the Bayesian Information Criterion (BIC). The best-fit scheme proposed two subsets: (*rps4-trnS*, *trnL-F*) and (*atpB*, *rbcL*). The best-fit model for subset1 was GTR+G and for subset2 was GTR+I+G. Maximum Likelihood analyses were performed using IQ-TREE v.1.6.8 (Nguyen et al. 2015) with 1000 thorough bootstrap replicates. Bootstrap values were labelled on the tree branches.

### Endangered categories analysis

Following the Red List Categories and Criteria (IUCN 2001), we used the GEOCAT tool (http://geocat.kew.org/; Bachman et al. 2011) to assess the current status of *Poromarattia tonkinensis*.

## Results

**Figure 2.**
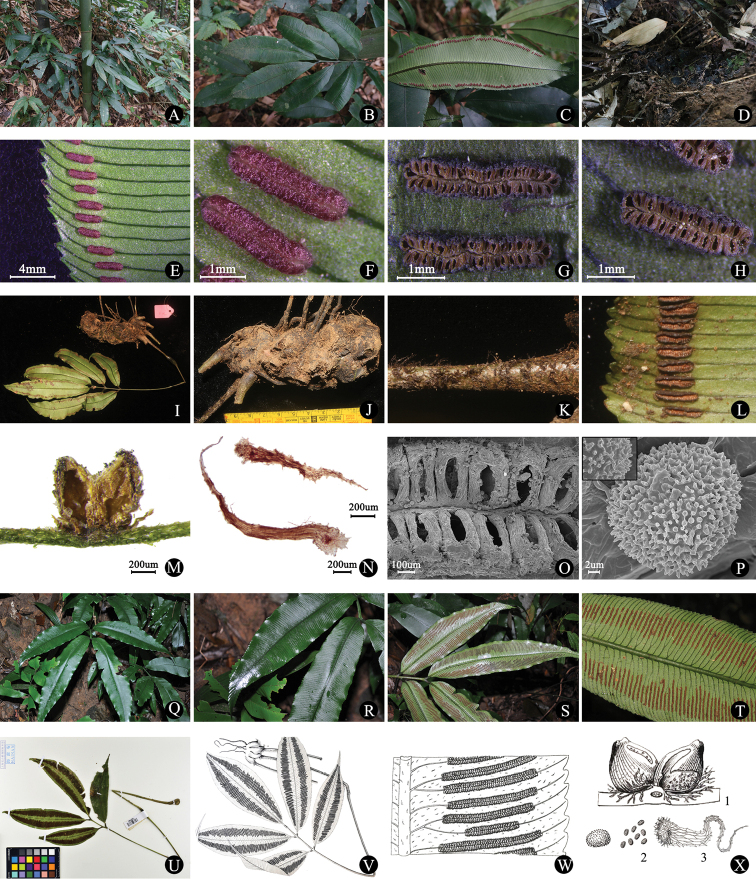
Morphological observation. Protomarattia tonkinensis (BX19001): **A** habit **B** frond **C, E, F, G, H, M, O** sporangia **D** rhizome **N** scale of stipe **P** exospores. Protomarattia tonkinensis (AT1-2): **I** whole plant **J** rhizome **K** stipe **L** sporangia. Archangiopteris tamdaoensis (SG2765): **Q, U** whole plant **R** frond **S, T** sporangia; Archangiopteris tamdaoensis (Ching 1958): **V** whole plant **W, X1** sporangia **X2** exospores **X3** scale.

The spores of *Protomarattia tonkinensis* were roundish-oblong. The ornamentation of the external perispore was coarsely echinate with spines occasionally forked at their apices and fused at their bases. Scales peltate, reddish-brown lanceolate, margin entire to sparsely denticulate, apex acuminate and scale cells elongate (Fig. 2). *Protomarattia tonkinensis* is morphologically quite similar to *Archangiopteris tamdaoensis* Hayata (Table 3), especially the horizontal dorsiventral rhizomes and simply pinnate fronds, but the former has submarginal synangia, ca. 4–6 mm, sporangia fully fused into synangia which lack pedicels, whereas the latter has medial sori, ca. 7–10 mm, sporangia respectively fused at base into receptacles (Hayata 1919; He and Christenhusz 2013).

**Table 3.** Morphological comparison of *Protomarattia tonkinensis* and *Archangiopteris tamdaoensis*.

Characters	*Protomarattia tonkinensis* Hayata (*BX19001* and *AT1-2*)	*Archangiopteris tamdaoensis* Hayata (*SG2765*; Hayata 1919; He and Christenhusz 2013)
Frond	20–30 cm	10–40 cm
Stipe	30–40 cm	40–45 cm
Rhizome	Long creeping	Long creeping
Scales of stipe	Reddish-brown lanceolate scales, with teeth on the edge.	Brown lanceolate scales, with teeth on the edge.
Pulvinus of stipe	1	1
Laminae	Once pinnate; pinnae 2 or 3 opposite or alternate pairs, elliptic. 25–28 cm × 5–6 cm.	Once pinnate; pinnae 2–4 alternate pairs, elliptic. 20–25 cm × 4–5 cm.
Veins	Obvious	Obvious
Sori	Synangium; locules have numerous sori; 3–5 mm from margin, ca. 4–6 mm.	Sporangia are independent of each other; medial between the costa and margin, 0.7–1 cm.
Exospores	Coarsely echinate	Rod-like ornamentation

**Figure 3.**
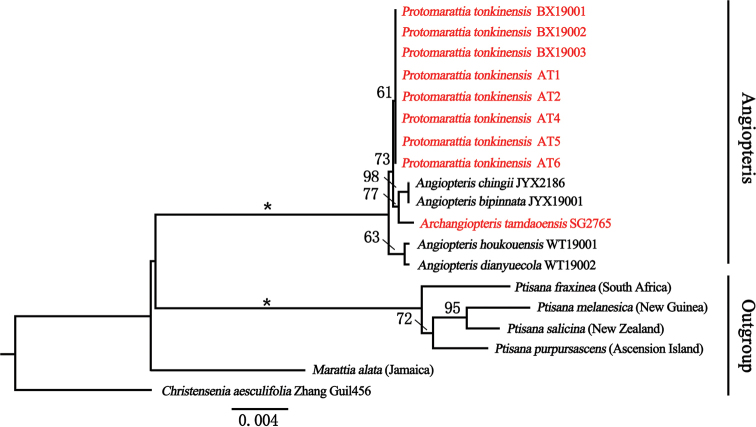
Maximum Likelihood phylogeny derived from the combined data (*atpB+rbcL*+*rps4-trnS*+*trnL-F*). Numbers on branches are support values of ML, * means 100%.

The combined data matrix included up to 2788 nucleotides for each of the 19 sequences of 12 species. The phylogenetic analyses (ML) resolved eight accessions of *Protomarattia tonkinensis* in a clade clearly separated from *Archangiopteris tamdaoensis* (Fig. 3). *Angiopteris*, *Archangiopteris* and *Protomarattia* form a monophyletic group with high support value, which proved that they were more closely related than with *Ptisana*.

## Discussion

In the field, the populations of *Protomarattia tonkinensis* have eight mature individuals in Malipo and seven mature individuals (clones of the same individual excluded) in Quan Ba. Before our study, the species had not been recorded since its publication about 100 years ago. The assessment result shows that *P. tonkinensis* should be assessed as Critically Endangered (CR). More fieldwork is needed in similar forest regions in south-western China, Myanmar and Vietnam to confirm its distribution and conservation status.

There have been a number of controversies surrounding *Protomarattia tonkinensis* since its publication. Some suggested that it should be treated as a synonym of *Archangiopteris tamdaoensis* (Christensen and Tardieu-Blot 1935; Copeland 1947; Ching 1958; Ching 1959; Chen 1964; Camus 1989; He and Christenhusz 2013), while others regarded *P. tonkinensis* as a “good” species and argued that *Protomarattia* should be treated as a different genus (Pichi-Sermolli 1968, Millay 1976). Based on morphological and phylogenetic analysis, we are supporting the decision by Camus (1989) to transfer *P. tonkinensis* to *Angiopteris tonkinensis* (Hayata) J.M.Camus. *Protomarattia tonkinensis*, however, is distinguishable from *Ar. tamdaoensis*, previously subsumed under *Angiopteris tonkinensis* by Camus (1989). We hereby propose a new combination *Angiopteris tamdaoensis* (Hayata) J.Y.Xiang & T.Wang, comb. nov.

### Taxonomic treatment

#### Angiopteris tonkinensis

Taxon classificationPlantaeMarattialesMarattiaceae

(Hayata) J.M.Camus

D88F4384-A459-5480-B8FB-29A0480FF917

Protomarattia tonkinensis Hayata, Bot. Gaz. 67: 88. 1919; Archangiopteris tonkinensis (Hayata) Ching, Ic. Fil. Sinic. V (1958) t. 209. Basionym

##### Type.

Vietnam. Tonkin, 30 July 1917, Bunzo Hayata *s.n.* (***Holotype***, K001057735!)

##### Additional specimens examined.

China. Yunnan: Zhuang-miao Autonomous Prefecture of Wenshan, Malipo, Hua mountain, Chouyang river, 850 m alt., 18 May 2018, J. Y. Xiang, T. Wang, M. F. Long, *BX19001* (SWFU); Vietnam. Ha Giang: Quan Ba, Thai An Commune, Seo Lung, 925 m alt., 10 October 2019, L. Averyanov, Nguyen Sinh Khang, T. Maisak, *AT1* (SWFU, HN).

##### Distribution.

Yunnan, China and Northern Vietnam.

#### Angiopteris tamdaoensis

Taxon classificationPlantaeMarattialesMarattiaceae

(Hayata) J.Y.Xiang & T.Wang comb. nov.

43472D22-6939-5243-BBAB-297CFCB99DDA

urn:lsid:ipni.org:names:77211596-1

Archangiopteris tamdaoensis Hayata, Bot. Gaz. 67: 88. 1919; Protangiopteris tamdaoensis (Hayata) Hayata, Bot. Mag. Tokyo 42(498): 309. 1928. Basionym

##### Type.

Vietnam Tonkin, August 1917, Bunzo Hayata, s.n.

##### Distribution.

Hainan, China and Northern Vietnam.

## Supplementary Material

XML Treatment for Angiopteris tonkinensis

XML Treatment for Angiopteris tamdaoensis
